# Factors Influencing the Outcome of Patients with Incidental Papillary Thyroid Microcarcinoma

**DOI:** 10.1155/2012/469397

**Published:** 2012-10-02

**Authors:** Beatriz Mantinan, Antonia Rego-Iraeta, Alejandra Larrañaga, Enrique Fluiters, Paula Sánchez-Sobrino, Ricardo V. Garcia-Mayor

**Affiliations:** ^1^Endocrine, Diabetes, Nutrition and Metabolism Department, University Hospital of Vigo, P.O. Box 1691, Plaza de Compostela 3, 36201 Vigo, Spain; ^2^Research Unit, University Hospital of Vigo, 36204 Vigo, Spain

## Abstract

*Objective*. To analyze some factors that could influence the outcome of patients with PTMC. *Material and Methods*. This is a longitudinal observational study. All patients diagnosed and treated for papillary thyroid microcarcinoma at the University Hospital of Vigo, between January 1994 and December 2003, were included in the present study. Demographic characteristics, tumour characteristics, TNM stage, rate of recurrence, and treatment with ^131^I were the study variables. *Results*. Ninety-one patients (75 females) with an average age of 47.7 ± 13.4 years, range 19–81, were studied. Initial tumour staging was T1 in 90 patients and T4a in 1 case. Initial lymph node involvement was present in 4 cases (4.4%). We only found one case with distant metastases at diagnosis. Postsurgical evaluation of thyroid specimens revealed that 28 (30.7%) tumours were multifocal. The average size of the tumour was 0.44 ± 0.25 cm, range 0.1–1. Univariate analysis reveals a statistically significant association between tumour multifocality and postsurgical ^131^I therapy with the recurrence rate. In the multivariate analysis only multifocality (*P* = 0.037, HR 5.7) was a significant risk factor for the recurrence rate. *Conclusions*. Our results indicate that tumour multifocality is an independent predictor of relapse but neither the tumour size nor postsurgical ^131^I therapy.

## 1. Introduction

Papillary thyroid microcarcinoma (PTMC) is defined as a tumour that measures of less than or equal to one centimetre in diameter [1]. It is often undetected in the clinical examination and found incidentally after thyroidectomy performed for other reasons or during a thyroid ultrasound exploration. 

In the latest TNM classification PTMC are included in T1 tumours, comprising tumours of up to two centimetres, without being considered as a specific entity in that classification [2].

Taking into account the presentation, PTMC can be divided into “incidentally detected” and “occult.” The term incidental refers to a tumour found during a thyroid surgical intervention for benign disease, during an autopsy, or during the performance of an ultrasound scan for another reason. Autopsy findings are frequent; some series reported a prevalence of up to 36% [3]. The average prevalence of PTMC is around 10% with a wide range 2.0–35.6% moreover mPTC patients represent up to 30% of all thyroid carcinomas [4–10]. The use of thyroid ultrasonography has led to an increase in the prevalence of PTMC. PTMC was defined as “occult” when it was undetected at clinical examination and indirectly diagnosed because of the presence of enlarged metastatic cervical lymph nodes or distant metastasis [11–16].

The clinical behaviour of these tumours tends to be indolent with an excellent prognosis. Most of the series have reported a very low mortality rate between 0% and 0.4% [11, 15, 17, 18]. Regardless of the presentation form of mPTC, multicentricity, bilaterality, and minor extrathyroidal invasion are found with the same frequency than clinical PTCs, while lymph node metastases and distal metastases occur with lower frequency [8, 10, 11, 15, 19–21]. 

The optimal management of PTMC patients can range from observation without surgery based on a recent clinical trial carried out by Japanese authors [22] to total thyroidectomy with neck dissection followed by radioiodine treatment depending on the presence of aggressive characteristics [16]. Some clinical and histological characteristics of patients with PTMC, such as age, sex, tumour size, histological features, multifocality, lymph node involvement, or distant metastases, have been proposed to stratify the patients with PTMC into high or low risk of recurrence [19–21]. Recently the tumour size has been gaining importance as a possible factor of poor prognosis [11, 19, 20]. 

 In the absence of consensus on some aspects of clinical management of patients with PTMC, we decided to do this work, with the aim of analyzing the features and long-term outcome of patients with PTMC diagnosed in our hospital over a 10-year period. We also assessed the factors associated with the recurrence rate, especially the influence of ^131^I adjuvant therapy. We hypothesized that tumour size, multifocality, and postsurgical ^131^I adjuvant therapy have influence on the rate of tumour recurrence.

## 2. Material and Methods

All patients were diagnosed and treated for PTMC, between January 1994 and December 2003 at the University Hospital of Vigo; they were 91 patients (75 females). The average age was 47.7 ± 13.4 years (range 19–81), with 39 (42.9%) patients being under the age of 45 years. The mean followup was 12.9 ± 4.9 years, ranging from 8 to 18 with a mean value of 10 years. The performed surgical procedure was total thyroidectomy in 72 cases (79.1%), total thyroidectomy with lymphadenectomy in 5 cases (5.5%) (In these cases the suspicious lymph nodes were observed during the surgical procedure.), and hemithyroidectomy in 14 cases (15.4%). With regard to the thyroid function, 80 (87.9%) of patients were euthyroid at diagnosis; the other 11 (12.1%) had thyroid hyperfunction (5 with Graves' disease and 6 with multinodular toxic goitre). 

The followup of patients was conducted until December 2011. The diagnosis of tumours was verified histologically by the Department of Pathology of our medical centre. For pathological examination, the whole thyroid gland was cut into 5 mm slices, fixed, and examined to define the dimensions of the malignant lesions and to note multifocality. Pathological slices were reviewed by an experienced pathologist. Vigo is located in North-Western Spain, a country without iodine deficiency [23]. All the cases included in the present study were classic histological variants of papillary thyroid carcinoma. Tumour staging was performed according to the sixth edition of “Cancer Staging Manual” [2]. During the period of enrolment of patients; we did not unify criteria for postsurgical ^131^I adjuvant therapy; some patients received this treatment and others did not, depending on the criteria of the doctor attended each patient. Postsurgical evaluation was performed every 3 to 6 months during the first year of followup and then every 12–18 months thereafter if patients were in remission of disease. All patients were treated with L-T4 with the aim of suppressing serum TSH, values (<0.3 mU/liter). Postoperative follow up included FT4, TSH and thyroglobulin determinations. Thyroglobulin and anti-thyroglobulin autoantibodies values were routinely determined in the same serum samples, only in cases of total or near total thyroidectomy. Also included ultrasonography and chest X-ray or CT scan.

### 2.1. Design

The present paper is an observational and longitudinal study. Almost all patients continued periodically going to our outpatient clinic. 

The local ethics committee approved the study protocol and all the patients signed the informed consent to participate in the study.

### 2.2. Study Variables

Demographic characteristics, age and gender, the purpose and mode of surgery, tumour characteristics such as tumour focality, tumour size, TNM stage, and rate of recurrence, and adjuvant treatment with ^131^I were the study variables.

### 2.3. Data Analysis

Continuous variables were expressed as means ± standard deviation, categorical variables as percentages. Differences were analyzed using *t*-test for continuous variables or if the distribution of the data was not normal using nonparametric *U* Mann-Whitney test. Chi-square or Fisher's exact test was used to compare categorical variables. The effects of each risk factor on DFS were evaluated using the Cox proportional hazards model. The level of statistical significance was set at *P* < 0.05. The statistical analyses were carried out using the SPSS v. 17.0 software package.

## 3. Results

Eighty-six cases (94.5%) were found during surgery of multinodular goitre (MNG) and 5 cases (5.5%) during surgery of Graves' disease. Initial tumour staging was T1 in 90 patients (98.9%) and T4a in 1 case (1.1%). Initial lymph node involvement was present in only 4 cases (4.4%). We only found one case (1.1%) with distant metastases at postsurgical evaluation.

Postsurgical evaluation of thyroid specimens revealed that 28 tumours (30.7%) were multifocal. The average size of the tumour was 0.44 ± 0.25 cm, range 0.1–1. Eight (8.8%) patients had local recurrent disease; the characteristics of patients with local recurrence are summarized in Table 1. The mean interval from diagnosis to recurrence was 4.2 ± 2.6 years. In the first postsurgical evaluation, 12 patients out of 77 (15.6%) who were treated with total thyroidectomy had positive values for antithyroglobulin autoantibodies; in 10 of them antithyroglobulin values became negative during the followup. Four patients died during followup, although none of the deaths were related to the tumour. 

Four patients had a history of cervical radiation therapy and revealed incidental findings during surgery for MNG with a single tumour focus, although none of them presented recurrence. 

Radioiodine ablation was administered to 45 patients (49.5%); 26 of them were treated for local or distant metastases. The average dose administered was 105.3 ± 42.2 mCi, range 63–230. The characteristics of the patients who received ^131^I treatment after surgery were similar to those who did not receive ^131^I except that the former group had high frequency of tumour multifocality (Table 2).

The univariate analysis reveals a statistically significant association between tumour multifocality and radioiodine treatment with the recurrence rate (Table 3). However, in the multivariate Cox analysis, only multifocality *P* = 0.037, HR 5.7, 95% CI: 1.107–29.4) was a significant risk factor for the recurrence rate in patient with PTMC (Table 4).

## 4. Discussion

Nonincidental papillary microcarcinomas have been excluded from the present study because they have a different clinical behaviour [10, 19, 24] with more frequent association to poor prognostic factors such as multifocality or capsular invasion and a higher rate of local or distant recurrence. 

The clinical significance of PTMC is still controversial. Its high prevalence in autopsy and as an incidental finding in thyroidectomy for benign pathology indicated its indolent course. The aggressiveness of the tumour refers to local or distant recurrences, which increases the morbidity due to more extensive surgery and presents a high rate of re-interventions. The reported tumour-related mortality ranges between 0 and 1% [4, 25]; in our work there is no case of death related to the tumour. 

Recently there is a trend to define a cut-off size above which the PTMC are more aggressive, defining it as local or distant recurrence. There is controversy regarding the cut-off point; Kasai and Sakamoto [26] distinguished small thyroid cancer <5 mm and ≥5 mm and suggested that this tumour size could have more aggressive clinical behaviour with an increased risk of lymph node metastasis. Roti et al. [19] found that tumours larger than 8 mm showed more aggressive behaviour defined as the presence of lymph node or distant metastases. Other series such as Chow et al. [11] and Wada et al. [20] found significant differences in the frequency of lymph node metastases among tumours larger than 5 mm compared with tumours less than 5 mm, although this difference does not impact significantly patient outcome. More recently, Besic et al. [27] found fewer recurrences in tumours less than or equal to 6 mm. Contrary to these studies, in the present one, we did not find that the tumour size significantly affects the rate of recurrence; differences with previous studies may be explained by the different methodological approaches. 

In other studies the factors that independently influence the rate of recurrence were lymph node involvement at diagnosis, extent of initial surgery [17], or tumour multifocality [15]. In our study the relapse rate observed (8.8%) is similar to the rate observed in other series [11, 17, 28]. We analysed the role of focality, tumour size, and TNM stage as a prognostic parameters of tumour recurrence. The univariate analysis showed significantly association between tumour multifocality and postsurgical ^131^I treatment with the relapse rate. This finding only is confirmed in the multivariate analysis with multifocality, being an independent risk factor for recurrence (HR 5.6).

There is no consensus regarding the treatment of PTMC; the spectrum ranges from observation without treatment [10, 25] to total thyroidectomy plus ablative therapy with ^131^I [19]. This disparity is explained by the lack of long-term epidemiological studies. The role of treatment with ^131^I after surgery is controversial because it is not clear if it reduces the relapse rate, which is already low in PTMC. In our centre there was no consensus regarding the recommendation of treating patients with PTMC with postsurgical radioiodine so only forty-five patients (49.5%) received postsurgical radioiodine treatment. The characteristics of the group of patients who received postsurgical radioiodine treatment were similar to the group that did not receive ^131^I therapy, except for the high frequency of tumour multifocality in the former group. The univariate analysis reveals a significantly higher recurrence rate in the group of patients treated with postsurgical radioiodine treatment that could be explained by the aforementioned high frequency of tumour multifocality. Interestingly seven of the eight patients who have presented local recurrence had received postsurgical radioiodine treatment; this is probably due to the presence of not radioavid lymph nodal metastases. This finding is in agreement with larger previous studies which have not found any benefit from using ^131^I therapy in patients with PTMC [29, 30]. 

## 5. Conclusions

In conclusion, our results indicate that tumour multifocality is an independent predictor of relapse but not of the tumour size in patients with PTMC. In general, the management of patients with PTMC should be in general conservative, in line with recent recommendations [22, 31]. Only in those cases having special characteristic, that is, aggressive variant of papillary tumour, multifocal tumours, locoregional extension or extrathyroidal invasion, the management should not differ from that of larger tumours.

## Figures and Tables

**Table 1:** Characteristics of the patients with local recurrence.

Patient number	Age (years)	Gender	^ 131^I therapy	Dose (mCi)	Tumour size (cm)	Focality	Side	TNM
1	40	Male	No	no	0.7	Unifocal	Right	T1NxM0
2	51	Female	Yes	120	0.3	Multifocal	Bilateral	T1NxM0
3	32	Female	Yes	240	0.3	Multifocal	Right	T1N1M0
4	53	Male	Yes	200	0.1	Unifocal	Left	T1N1M1
5	44	Male	Yes	160	0.5	Unifocal	Right	T1NxM0
6	45	Male	Yes	100	0.4	Multifocal	Left	T1N1M0
7	44	Female	Yes	80	0.2	Multifocal	Left	T1NxM0
8	23	Female	Yes	100	0.5	Multifocal	Left	T1NxM0

**Table 2:** Comparison between the main characteristics patients who received ^131^I therapy and those who did not.

	^ 131^I therapy (*N* = 45)	No ^131^I therapy (*N* = 46)	*P*
Age (years)	46.07 ± 12.07	49.35 ± 14.5	0.54
Male gender (%)	20	15.2	0.59
Tumour size (cm)	0.48 ± 0.24	0.41 ± 0.24	0.71
Multifocality (%)	48.9	13	**0.04**
T1 with capsular invasion (%)	2.2	0	0.49
Ganglionar disease (%)	6.7	2.2	0.36
Distant metastasis (%)	2.2	0	0.48

**Table 3:** Comparison between the main characteristics of patients without or with tumour recurrence.

	Patients without recurrence (*n* = 83)	Patients with recurrence (*n* = 8)	*P*
Age (years)	48.32 ± 13.6	41.53 ± 10	0.10
Male gender (%)	15.7	37.5	0.14
Total thyroidectomy (%)	84	87.5	0.11
Tumour size (cm)	0.45 ± 0.25	0.37 ± 0.19	0.29
Tumour multifocality (%)	27.7	62.5	**0.043**
T1 with capsular invasion (%)	1.2	0	0.75
Lymph nodal metastases N1 (%)	4.8	0	0.52
Distant metastasis (%)	0	12.5	0.088
^ 131^I therapy (%)	45.8	87.5	**0.03**

**Table 4:** Cox regression analysis of predictive variables.

	*B*	OR	95% CI	*P*
Tumour multifocality	1.7	5.7	1.10–29.4	**0.037**
^ 131^I therapy	−1.07	0.34	0.033–3.56	0.36
Tumour size	−0.30	0.73	0.022–25.09	0.86
